# Coronary computed tomography angiography in the evaluation of acute chest pain in patients with elevated high sensitive cardiac troponin I (hs-cTn) level

**DOI:** 10.1016/j.ahjo.2023.100276

**Published:** 2023-02-17

**Authors:** Majd Qasum, Samia Massalha, Erez Marcusohn, Adi Elias, Said Darawshi, Robert Zukermann

**Affiliations:** a Departments of Cardiology, Rambam Health Care Campus, Haifa, Israel; b Department of Nuclear Medicine, Rambam Health Care Campus, Haifa, Israel; c Department of Medicine D, Ruth & Bruce Rappaport Faculty of Medicine, Rambam Health Care Campus, Technion-IIT, Haifa, Israel

**Keywords:** Acute coronary syndrome, High sensitivity cardiac troponin, Coronary computed tomography angiography, Non-ST elevation myocardial infarction

## Abstract

Aims CCTA is a well-established and safe imaging modality for the diagnosis of CAD and is gate keeping for invasive coronary angiography (ICA). We aimed to examine CCTA performance in patients presenting with ACP and dynamic hs-cTn elevation compatible with MI but not exceeding 7 folds of the URL. We also examined the performance of GRACE and PTP consortium scores in this population of patients.

## 1 Introduction

Acute chest pain (ACP) is one of the most common complaints of patients presenting to the emergency department (ED) [1], [2]. ACP is suspected as acute coronary syndrome (ACS) based on clinical assessment, elevation of myocardial damage biomarkers and ST-T changes on ECG. In some cases, with clinical suspicion of ACS without ECG changes suggestive of ACS, the diagnosis is based on risk stratification and cardiac troponin elevation [3], [4], [5], [6], [7]. As cardiac troponins elevation can be found in other non-cardiac etiologies, the evaluation of suspected ACS remains a clinical challenge.

The latest ESC Guidelines recommend coronary computed tomography angiography (CCTA) as an alternative to invasive coronary angiography (ICA) to exclude ACS in patients with low-to-intermediate likelihood for the presence of coronary artery disease (CAD), normal or *inconclusive* cardiac troponin and/or ECG without ischemic changes [8].

CCTA was found to have a high negative predictive value in (>99.5 %) in excluding ACS in several recent clinical trials [9], [10]. Furthermore, CCTA effectively excludes other causes of ACP that could be associated with high morbidity and mortality, mainly pulmonary embolism, aortic dissection and tension pneumothorax [8].

In the recent years hs-cTn measurements have become a standard test in the management of patients presenting with ACP with both high negative and positive predictive value for diagnosis of myocardial infarction (MI) [11], [12], [13]. Hs-cTn assays have also been found to be elevated in patients with non-cardiovascular and non-coronary artery disease [14], [15], [16], [17]. Therefore, there is an increased prevalence of ICA with no evidence of obstructive coronary artery disease [18].

According to ESC guidelines, patients with clinical presentation indicative of MI and a dynamic elevation of hs-cTn above upper reference limit (URL) have a high risk for adverse events, even though an elevation up to 3-fold the URL has only limited (50–60 %) positive predictive value (PPV) for acute MI and may be associated with a broad spectrum of cardiac and non-cardiac conditions [8].

There are several clinical scores available which are intended to predict the probability of CAD, risk stratification and to guide treatment of patients, e.g. GRACE risk score, TIMI score, HEART score and the PTP consortium score. Among these, the GRACE risk score offers the best discriminative performance and is recommended to estimate prognosis according to the recent ESC guidelines [8], and it was found to be superior when compared to TIMI score in estimation of 1-year endpoint of death or MI [19]. The HEART score is aimed to identify patients presenting to the ED with intermediate or high risk for adverse outcome and supports medical decision to admit patients for further evaluation, this score isn't suitable for our study population as it will be at least moderate in the vast majority of patients due to the elevated hs-cTn level and the presence of chest pain and cardiovascular risk factors. Another available risk score is the PTP consortium risk score which is validated for patients with stable chest pain for predicting the probability of CAD.

We hypothesize that CCTA is a safe alternative to ICA for the initial evaluation of patients presenting with suspected ACS and have mild hs-cTn level elevation (up to 7 folds of URL), in addition to its ability to lower the need for invasive evaluation in these patients.

We aimed also to examine the performance of risk scores, such as the GRACE risk score and the PTP consortium score.

To the best of our knowledge this is the first study to examine the potential of using CCTA to exclude CAD in patients presenting to ED with ACP and mildly elevated hs-cTn and it's also the first to evaluate the performance of PTP consortium risk score in this population of patients.

## 2 Methods

### 2.1 Study design and data sources

A retrospective cohort included patients admitted to the hospital for the investigation of ACP and had a dynamic elevation of hs-cTn levels compatible of MI who underwent CCTA as the initial evaluation test for suspected ACS between February 2016 and October 2020. Patients were included in the analysis only if ACP was the primary symptom for which they presented to the ED and had a hs-cTn I level between 30 and 210 ng/l. All patients were older than 18 years.

All records were reviewed by a senior cardiologist using the electronic medical records to determine which patients will be included in the final analysis. CCTA was performed using the Philips 128 cardiac CT scanner.

Patients were excluded if they had: a previous diagnosis of ischemic heart disease (PCI or CABG in the past), heart failure with reduced ejection fraction (HFrEF), hypertrophic cardiomyopathy (HCM), end stage renal disease (ESRD), atrial or ventricular arrhythmia as the cause of the admission and patients with suspected type II myocardial infarction (MI) at presentation as defined in ESC guidelines [20].

The hs-cTn I test was performed using the ARCHITECT® Abbott assay.

In the general population the 99th percentile of this assay has been reported at 30 ng/l.

We chose our reference level to be above 99th percentile of the upper reference limit (URL) and up to 7-folds of the URL in order to avoid including patients with very high risk for death or complications.

Pre-test probability of CAD (CAD consortium) score and GRACE risk score were calculated for all patients. The Pre-test probability of CAD (CAD consortium) score model uses patient data including age, sex, symptoms, and cardiovascular risk factors to allow for accurate estimation of the pretest probability of CAD in low prevalence populations. Low PTP score is defined as a score below 50 % and high PTP score is defined as ≥50 % [21]. Even though this model was validated to determine the pre-test probability in patients with stable chest pain, we aimed to examine its performance in patients with ACP. Regarding GRACE score, Low risk score is defined as a score ≤ 108, intermediate risk >108 and ≤140, and high risk >140.

### 2.2 Dependent variable

1. Primary outcome: Patients proceeding to coronary revascularization (PCI or CABG).
2. Secondary outcome: Patients proceeding to invasive diagnostic catheterization.

### 2.3 Outcome measures and variables

The following data were retrieved from the electronic medical records of the patients:

1. Demographics: age, gender;
2. Vital signs at admission: heart rate, systolic and diastolic blood pressure;
3. Comorbidities: hypertension, diabetes mellitus, hyperlipidemia, obesity, Tobacco use, arrhythmias, valvular heart disease, cerebrovascular disease, prior PCI or CABG;
4. Patient's main complaint.
5. Lab data: hs-cTn maximal level and creatinine at admission;
6. CCTA results as normal/non-significant CAD, or significant CAD.
7. For patients proceeding to invasive evaluation: diagnostic procedure or type of revascularization by PCI or CABG;

### 2.4 Statistical analysis

Normally distributed variables presented as mean ± standard deviation. Categorical variables presented as numbers and percentages. Baseline characteristics of groups compared using the unpaired *t*-test for continuous variables and the Chi-squared test for categorical variables.

Variables that are associated with increased risk of proceeding to PCI or CABG in the univariable analysis (*P* < 0.2) were included in the multivariable model. Logistic regression with backward LR used to assess predictors of proceeding to PCI or CABG.

The strength of the association of the predictors were estimated with odds ratio (OR) and 95 % confidence interval (CI).

The calibration of the model assessed using the *Hosmer*-*Lemeshow* goodness of fit test. The discrimination of the model assessed by calculating the area under the curve (AUC) of the receiver operating characteristic (ROC) curve.

Differences were considered statistically significant at the 2-sided *P* < 0.05 level. Statistical analyses performed using SPSS version 26 ((IBM Corp. Armonk, New York)).

## 3 Results

**Fig. 1:**
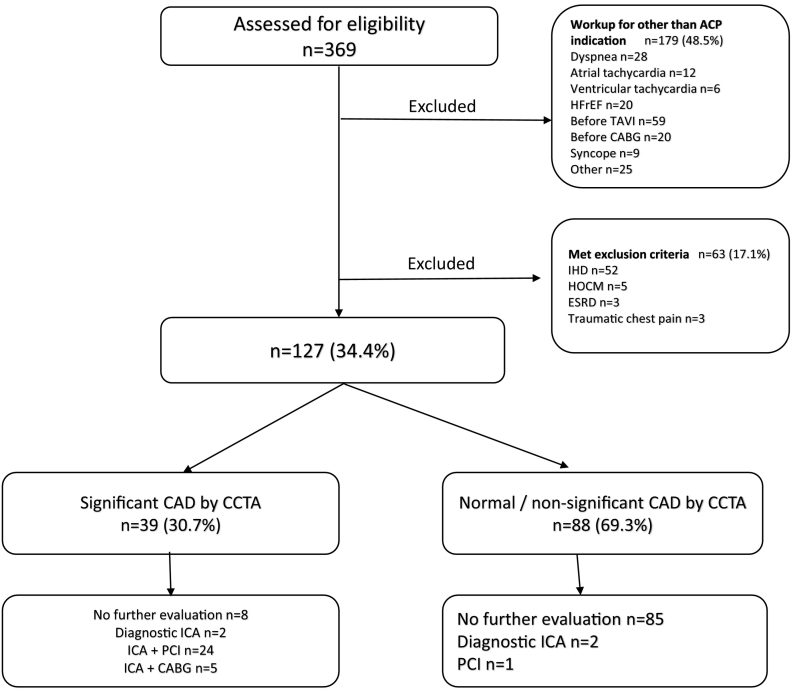
HFrEF: heart failure with reduced ejection fraction; TAVI: trans-catheter aortic valve implantation; CABG: coronary artery bypass graft; IHD: ischemic heart disease; HOCM: hypertophic obstructive cardiomyopathy; ESRD: end stage renal disease; CAD: coronary artery disease; ICA: invasive coronary angiography; PCI: percutaneous coronary intervention.

During the study period, a total of 369 patients with hs-cTn level ranging between 30 and 210 ng/l underwent CCTA. One hundred and twenty-seven patients had a primary complaint of ACP and met all the inclusion criteria. Thirty-nine patients (30.7 %) had significant CAD (>70 % stenosis in ≥1 major epicardial artery or >50 % in the left main stem) by CCTA, 31 patients (24.4 %) underwent ICA and ultimately 29 patients (22.8 %) proceeded to revascularization by PCI or CABG. Eight patients (6.3 %) with significant CAD didn't undergo further evaluation based on patient's choice or due to borderline CAD. Additionally, three patients with normal/non-significant CAD by CCTA underwent ICA and ultimately only one patient needed revascularization by stent implantation due to significant distal stenosis. At one year follow up, none of the patients with non-significant CAD underwent ICA (Fig. 1).

The baseline demographic and clinical characteristics are summarized in Table 1, Table 2. 106 patients (83 %) were classified as low risk (GRACE risk score ≤ 108), while 21 patients (17 %) were classified as moderate risk (GRACE risk score > 108–140). There were no statistically significant differences between the groups regarding cardiovascular risk factors, findings on CCTA, ICA results or need for revascularization.

**Table 1:** Baseline characteristics by GRACE score.

Variable	Total	GRACE≤108	GRACE>108-≤140	P value
Number of patients	127	106 (83.46 %)	21 (16.54 %)	
Age, y (SD)	57.2 ± 13.35	53.53 ± 11.26	75.67 ± 5.3	<0.001
Female sex	57 (44.9 %)	44 (41.5 %)	13 (61.9 %)	0.086
Diabetes	54 (42.5 %)	45 (42.5 %)	9 (42.9 %)	0.973
Hypertension	101 (79.5 %)	84 (79.2 %)	17 (81 %)	0.859
Hyperlipidemia	93 (73.2 %)	79 (74.5 %)	14 (66.7 %)	0.457
Obesity	26 (20.5 %)	22 (20.8)	4 (19 %)	0.859
Smoking	37 (29.1 %)	30 (28.3 %)	7 (33.3 %)	0.643
Atrial arrhythmia	11 (8.7 %)	4 (3.8 %)	7 (33.3 %)	<0.001
GRACE score (SD)	83.91 ± 22.21	77 ± 16	121 ± 9	0.001
Pre-test probability ≥50 %	42 (33 %)	27 (25.5 %)	15 (71.4 %)	<0.001
Maximal troponin (ng/l)	78 ± 125.6	77 ± 129	83 ± 110	0.0854
Significant CAD by CCTA	39 (30.7 %)	32 (30.2 %)	7 (33.3 %)	0.367
Revascularization by PCI/CABG	30 (23.6 %)	24 (22.6 %)	6 (28.5 %)	0.432

Data are given as mean ± SD, number (percentage), or median (interquartile range).

**Table 2:** Baseline characteristics by pre-test probability.

Variable	Total	PTP < 50 %	PTP ≥ 50 %	P value
Number of patients	127	85	42	
Age, y (SD)	57.2 ± 13.35	52.6 ± 12.61	66.33 ± 9.7	0.001
Female sex	57 (44.9 %)	47 (55.3 %)	10 (23.8 %)	0.001
Diabetes	54 (42.5 %)	28 (32.9 %)	26 (61.9 %)	0.002
Hypertension	101 (79.5 %)	63 (74.1 %)	38 (90.5 %)	0.032
Hyperlipidemia	93 (73.2 %)	60 (70.6 %)	33 (78.6 %)	0.339
Obesity	26 (20.5 %)	19 (22.4 %)	7 (16.7 %)	0.455
Smoking	37 (29.1 %)	16 (18.8 %)	21 (50 %)	<0.001
Atrial arrhythmia	11 (8.7 %)	4 (4.7 %)	7 (16.7 %)	0.024
Troponin admission mean (SD)	78 ± 125.6	79 ± 140	76 ± 89	0.89
Maximal troponin (ng/l), median [IQR]	59 [41.5–128.5]	58 [41–112]	70 [42–142]	0.32
Significant CAD by CCTA	39 (30.7 %)	16 (18.8 %)	23 (54.8 %)	0.001
Revascularization by PCI/CABG	30 (23.6 %)	14 (16.5 %)	16 (38.1 %)	0.007

Data are given as mean ± SD, number (percentage), or median (interquartile range).

When dividing into two groups according to PTP of CAD score (CAD consortium) which is based on patients' age, sex, chest pain characteristics and CV risk factors (diabetes, hypertension, dyslipidemia and smoking history), patients with intermediate-high PTP (≥50 %) were older, more likely to be males and with diabetes mellitus, hypertension and smoking history (Table 1). In the group with low PTP score 16 (18.8 %) patients had significant CAD and 23 (54.8 %) patients in the intermediate-high risk score (*P* value 0.001). Eventually, 14 (16.5 %) patients underwent revascularization (by PCI or CABG) in the low risk group and 16 (38.1 %) in moderate-high risk group (*P* value 0.007) (Table 2).

On univariate analysis, smoking and moderate to high PTP score were variables associated with the high likelihood of revascularization in this cohort of patients (Table 3).

**Table 3:** Univariate analysis for predictors of eventually proceeding to PCI or CABG.

Variable	PCI or CABG	No intervention	P value
Number of patients	30	97	
Age, y	58.6 ± 11.48	56.75 ± 13.9	0.5
Female sex	9 (30.0 %)	48 (49.5 %)	0.061
Diabetes mellitus	16 (53.3 %)	38 (39.2 %)	0.17
Hypertension	26 (86.7 %)	75 (77.3 %)	0.267
Hyperlipidemia	23 (76.7 %)	70 (72.2 %)	0.62
Obesity	3 (10.0 %)	23 (23.7 %)	0.1
Smoking	13 (43.3 %)	24 (24.7 %)	0.05
Atrial fibrillation/atrial flutter	2 (6.7 %)	9 (9.3 %)	0.65
GRACE score	86 ± 21	83 ± 23	0.628
Pre-test Probability ≥50 %	16 (53.3 %)	26 (26.8 %)	0.007

Multivariable analysis that included variables with P value below 0.2 in the univariate analysis were included in the final model and demonstrated increased risk of revascularization in patients with PTP ≥ 50 % with odds ratio (OR) 3.439 (95 % confidence interval (CI), 1.424–8.302) (Table 4). The final model had a good discriminative ability, with AUC 0.736.

**Table 4:** Multivariable model.

Variable	Odds ratio (95 % CI)	P value
Variables entered to the model		
Gender	0.6 (0.219–1.647)	0.322
Diabetes mellitus	1.746 (0.669–4.556)	0.255
Obesity	0.362 (0.091–1.436)	0.362
Smoking	2.153 (0.713–6.505)	0.1740
Maximal troponin	0.988 (0.977–0.999)	0.036
Pre-test probability ≥50 %	2.299 (0.842–6.275)	0.104

Final model		
Maximal troponin	0.987 (0.975–1.00)	0.024
Pre-test probability ≥50 %	3.439 (1.424–8.302)	0.006

Finally, CCTA demonstrated very good diagnostic accuracy for the need for revascularization, the sensitivity, specificity, PPV and NPV were 96.7 %, 89.7 %, 74.4 % and 98.9 % respectively.

## 4 Discussion

The evaluation of patients presenting with ACP and suspected non-STEMI is constantly improving, alongside the clinical presentation it's currently based on hs-cTn measurement which has both a high negative and positive predictive values for diagnosis of MI. Advances in technology and refinement in cardiac troponin assays have improved the ability to detect and quantify cardio-myocyte injury. These hs-cTn assays allow for rapid ‘rule-in’ and ‘rule-out’ of MI based on evidence that elevation beyond 5-fold of the URL have a high PPV (>90 %) for acute MI [8].

While hs-cTn assays are sensitive to myocardial injury, on clinical grounds it may be difficult to distinguish the dynamic troponin changes caused by a NSTEMI versus other cardiac or non-cardiac conditions causing myocardial injury [22]. The ESC guidelines adapted CCTA as an alternative to ICA to exclude ACS in patients with low-to-intermediate likelihood of CAD with inconclusive cardiac troponin [8]. In clinical practice this statement may be misleading since hs-cTn assays as opposed to conventional assays are more sensitive to myocardial injury which may indicate myocardial injury secondary to microvascular damage, type II MI or non-cardiac pathologies, and not by plaque rupture or thrombosis [23]. Usually, these patients are treated as having MI and are referred to ICA, even though up to one-third of these patients do not have an obstructive coronary artery disease as shown by one study [24].

CCTA proved its importance in triage of patients presenting with ACP and normal cardiac troponin [25] with high negative predictive value (>99.5 %) to exclude ACS [8], [26]. The recommendation of CCTA as diagnostic tool for patients with low to intermediate risk without signs of ischemia on ECG and normal cardiac troponin is based on several clinical trials. However, the majority of these trials used only conventional troponin assays that are less sensitive, therefore its application for patients presenting with moderately elevated hs-cTn remains unknown. Nevertheless, in patients with low-to-intermediate likelihood of CAD with inconclusive troponins levels, CCTA can rule out obstructive CAD and can differentiate between coronary, myocardial and non-cardiovascular etiologies [25], [27], [28].

In the present study we did not find any difference between the low-intermediate vs high risk group based on GRACE score. Although the group with low-intermediate GRACE score had significantly higher PTP score than the low-risk group, without any difference in atherosclerotic risk factors, there were no differences between groups regarding the findings on CCTA (significant CAD, 30.2 % vs 33.3 %, respectively, p = NS) nor the ICA results or the need for revascularization (PCI and/or CABG, 22.6 % vs 28.5 % respectively, p = NS). These findings suggest that the GRACE score is a poor risk stratification tool in patients with ACP and mildly elevated hs-cTn I level, as this risk score did not predict significant CAD on CCTA in this cohort of patients with inconclusive elevation of hs-cTn or predict patients who will require revascularization.

Smulders et al. found that 34 % of patients with ACP and elevated hs-cTn levels who underwent CCTA as first line imaging modality did not have significant CAD and concluded that in those patients CCTA can be novel strategy imaging other than ICA [29]. In contrast, other study that compared diagnostic strategy by early CCTA with standard of care encompassing high-sensitivity hs-cTn for patients suspected of ACS found that the total number of ICAs performed within 30 days and the number of patients requiring revascularization was the same between the CCTA group and standard of care group [22]. In a recent meta-analysis that compared the efficacy and clinical outcomes between CCTA-based strategy and other standard of care approaches in patients with ACP, CCTA was associated with similar major adverse cardiac events but higher rates of revascularization [30].

In this study, GRACE risk score and CAD consortium clinical PTP scoring system were calculated for all patients. The GRACE risk score failed to predict the presence of significant CAD or to predict patients who will require revascularization. However, the PTP consortium clinical risk score, which is an updated version of the Diamond-Forrester model, did predict both parameters. There were significant differences between both methods, patients with the higher PTP were significantly older, more likely to be males, with diabetes mellitus, hypertension and smoking. There was no difference regarding hs-cTn level. On multivariate analysis, PTP score (≥50 %) was a strong predictor for coronary revascularization with odds ratio of 3.439 (CI 1.424–8.302).

In the CATCH trial, most patients had low to intermediate PTP score with significant reduction in MACE with a CCTA-guided strategy compared to a strategy of standard functional testing [31]. Although this model was not validated in patients with suspected ACS, we found in our cohort of patients that PTP score did predict both: significant CAD on CCTA and the need for revascularization.

Finally, in our cohort of patients, CCTA demonstrated very good diagnostic accuracy for the need for revascularization; the sensitivity, specificity, PPV and NPV were 96.7 %, 89.7 %, 74.4 % and 98.9 % respectively.

In summary, our study results suggest that in patients with ACP and mild hs-cTn elevation, PTP consortium risk score may have an additional value in the decision of evaluating patients with CCTA or ICA, as patients with low PTP score can be evaluated with CCTA as the first line modality of imaging due to the low prevalence of significant CAD and the high NPV of the CCTA. On the contrary, for patients with high PTP risk score, CCTA testing is unnecessary and direct invasive evaluation may be warranted, as the probability of significant CAD is high. Larger scale prospective studies are needed.

## 5 Limitations

The present study is retrospective in nature, there was no control group for patients who underwent cardiac catheterization directly without prior evaluation by CCTA.

Another limitation is the number of patients who were evaluated, which is limited because most patients undergo ICA directly without any further evaluation in-line with current guidelines and a larger scale prospective study is needed in order to change clinical practice.

The primary outcome was limited to significant/non-significant CAD and whether the patient underwent revascularization or not.

The number of revascularizations might be underestimated, since eight patients with significant CAD by CCTA didn't undergo coronary angiography.

## Declaration of competing interest

The authors declare that there is no conflict of interest that could be perceived as prejudicing the impartiality of the research reported.
